# Dose‐ and Duration‐Dependent Effects of Propylene Glycol on Lipid Metabolism‐Related mRNAs, Proteins, and Fatty Acids in the Adipose Tissue of Fattening Akkaraman Lambs

**DOI:** 10.1002/fsn3.71336

**Published:** 2025-12-10

**Authors:** Akın Yakan, Hüseyin Özkan, Hasan Hüseyin Keçeli, Ufuk Kaya, Irem Karaaslan, Sevda Dalkiran, Necmettin Ünal, Baran Çamdeviren, Güven Güngör, Mehmet Küçükoflaz, Savaş Sariözkan, Korhan Arslan, Bilal Akyüz, Aytaç Akçay, Ceyhan Özbeyaz

**Affiliations:** ^1^ Department of Genetics, Faculty of Veterinary Medicine Hatay Mustafa Kemal University Antakya Hatay Turkey; ^2^ Department of Biostatistics, Faculty of Veterinary Medicine Hatay Mustafa Kemal University Antakya Hatay Turkey; ^3^ Technology and Research & Development Center (MARGEM), Hatay Mustafa Kemal University Antakya Hatay Turkey; ^4^ Department of Molecular Biochemistry and Genetics, Institute of Health Sciences Hatay Mustafa Kemal University Antakya Hatay Turkey; ^5^ Department of Animal Science, Faculty of Veterinary Medicine Ankara University Ankara Turkey; ^6^ Department of Biostatistics, Faculty of Veterinary Medicine Bingöl University Kayseri Turkey; ^7^ Department of Animal Health Economics and Management, Faculty of Veterinary Medicine Kafkas University Kars Turkey; ^8^ Department of Animal Health Economics and Management, Faculty of Veterinary Medicine Erciyes University Kayseri Turkey; ^9^ Department of Genetics, Faculty of Veterinary Medicine Erciyes University Kayseri Turkey; ^10^ Department of Biostatistics, Faculty of Veterinary Medicine Ankara University Ankara Turkey

## Abstract

Propylene glycol (PG) is incorporated into ruminant diets to boost glucogenic energy availability, yet its precise effects on adipose tissue development remain incompletely defined. The study was designed as a 3 × 3 factorial experiment with two independent variables: dose of PG and duration of fattening. Three groups were formed, including a dose group of PG 1.5 mL/kg live weight (PG1.5), a dose group of PG 3 mL/kg live weight (PG3), and a group without PG (PG0). Gluteal adipose tissues were collected from animals slaughtered on days 60, 90, and 120. mRNA, protein, and fatty acid profiles were analyzed. Protein–protein interaction and gene set enrichment analysis were also performed. On day 60, FABP4 was approximately 3‐fold higher at both mRNA and protein levels in PG3 compared to PG0, nearly 2‐fold higher at the protein level in PG1.5, and SREBP‐1c protein levels were reduced in PG1.5 compared to PG0. On day 120, FABP4, PPARγ, C/EBPα exhibited an increasing trend at both mRNA and protein levels in PG groups, whereas SREBP‐1c was decreased in PG3. Fatty acid profiling revealed C16:0, C18:0, and C18:1 comprised over 70% of total lipids. PG supplementation shifted the profile toward unsaturated species, reducing saturated fatty acid proportions and enhancing nutritional indices, particularly in PG1.5. Findings at the bioinformatics levels demonstrate PG exerts clear dose‐ and time‐dependent modulation of adipogenic transcription factors, fatty acid composition, and molecular interaction networks in lamb adipose tissue. Early PG3 feeding elevates FABP4 and suppresses SREBP‐1c, whereas prolonged supplementation enhances PPARγ and C/EBPα and drives a favorable shift in lipid profiles. Network and pathway analyses reveal coordinated regulation via NR1H3/RXR and PPAR axes, suggesting PG not only optimizes energy partitioning but also supports cellular homeostasis. These results could contribute to the development of potential strategies aimed at supporting adipose tissue quality and metabolic health in sheep.

## Graphical abstract

Propylene glycol modulated lipid metabolism in gluteal fat tissue. Expression of FABP4 at Day 60 and 120, and PPARγ and C/EBPα at Day 120 were upregulated, while SREBP1c was downregulated at both the mRNA (Day 120) and protein levels (Day 60 and 120). These results indicate that propylene glycol enhances lipid mobilization and reduces lipogenesis through the transcriptional regulation of key adipogenic markers.

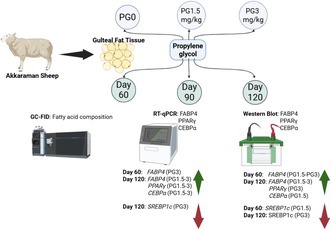

## 1 Introduction

Sheep breeding, particularly focused on meat production, is a significant aspect of livestock production. In addition to meat yield, secondary traits such as adipose tissue are also of considerable importance in farm animals. Adipose tissue serves as a central component of energy metabolism in the organism. During the fattening period, increases in muscle mass and adipose tissue content are observed depending on the energy content of the diet (Chikwanha et al. 2018; Yakan et al. 2024; Ateş et al. 2025).

In ruminant farming, various strategies are employed to enhance growth rate and fattening performance, with a primary focus on optimizing diet composition and energy content (Yakan et al. 2024). Propylene glycol (Propane‐1,2‐diol), categorized as a food additive under the European Union Food Codex, is a glucogenic compound widely used in ruminants to mitigate energy deficits, treat ketosis, and enhance milk production (Merck Index 1989; Chiofalo et al. 2005; Jeong et al. 2018; van der Drift et al. 2015).

Adipose tissue, being metabolically active, plays a pivotal role in energy metabolism. The molecular regulation of energy metabolism and fatty acid profiles in adipose tissue is critical for lamb growth, production traits, and quality parameters (Ateş et al. 2025).

This study investigates the effects of different doses and durations of propylene glycol supplementation during the growth period in Akkaraman lambs on energy metabolism and lipogenesis pathways within gluteal adipose tissue. The study specifically evaluates the mRNA and protein levels of major regulators, including FABP4 (Fatty Acid Binding Protein 4), C/EBPα (CCAAT/enhancer‐binding protein alpha), SREBP‐1c (Sterol Regulator Element Binding Protein 1c), and PPARγ (Peroxisome Proliferator Activated Receptor Gamma), as well as the fatty acid profile in the tissue.

## 2 Materials and Methods

The study was conducted at the Small Ruminant Experiment Unit, licensed for Animal Use in Experimental Research by both the Erciyes University Agricultural Research and Application Center (ERUTAM) and the Republic of Türkiye Ministry of Agriculture and Forestry. The experimental protocol was ethically approved by the Erciyes University Animal Experiments Local Ethics Committee (06.03.2019, 19/044).

The study material consisted of 72 male Akkaraman lambs, weaned at 2–3 months of age, weighing 15–20 kg, and purchased from ERUTAM. The study was designed as a 3 × 3 factorial experiment with two independent variables: the dose of propylene glycol (PG) and the duration of the fattening period. Three groups were formed, including a dose group of PG 1.5 mL/kg live weight^0.75^ (PG1.5), a dose group of PG 3 mL/kg live weight^0.75^ (PG3), and a without PG (PG0) group. The dose calculation was done according to (Kim et al. 2005). The lambs were divided into three groups that were slaughtered after 60, 90, and 120 days of receiving PG (Yakan et al. 2024).

The lambs were housed in groups, with a total of 18 pens accommodating 4 lambs each (4 × 18 = 72 lambs). Each treatment combination was assigned to six pens, with two pens allocated per Dose × Feeding Group. Each pen measured 6.6 m^2^, comprising a 3 m^2^ enclosed area and a 3.6 m^2^ open area, providing the lambs with unrestricted access to both spaces. In the study, the lambs were provided ad libitum access to clean water and a commercial lamb growth feed containing 2600 kcal/kg ME and 16% crude protein (CP), formulated according to the nutrient requirements for growing lambs reported by the NRC (2007). Additionally, each lamb was supplied with 100 g/day of roughage in the form of dry alfalfa.

The lambs were slaughtered on the 60th, 90th, and 120th days of the feeding period. On each slaughter day, eight lambs from each group (PG0, PG1.5, and PG3) were processed. The animals were slaughtered at the abattoir located within the same institutional complex as the feeding units. Following slaughter, small sections of gluteal fat tissues (GFTs) were carefully collected under nuclease‐free conditions and immediately snap‐frozen in liquid nitrogen for subsequent gene expression and protein analyses. The collected tissues were stored at −80°C until molecular analyses.

### 2.1 Fatty Acid Analysis

Fat extraction was carried out using the Diethyl Ether method (Ekiz et al. 2019), followed by the conversion of fatty acids into their methyl ester derivatives. These methyl esters were analyzed using a Gas Chromatography system equipped with a flame ionization detector (Shimadzu GC‐2025, Japan), an automatic injector (Shimadzu AOC‐20i, Japan), and a Restek Rt‐2560 column (100 m × 0.25 mm ID × 0.20 μm). The instrument settings were as follows: injector and detector temperatures set at 250°C, carrier gas flow rate maintained at 1.20 mL/min, and an injection volume of 1 μL. Hydrogen served as the carrier gas. A temperature gradient program was applied, starting with an initial column oven temperature of 100°C held for 2 min, then increased at a rate of 4°C/min until reaching 250°C, where it was held for 15 min. Fatty acid identification was confirmed by comparing the chromatographic peaks with an internal standard (FAME Mix, Restek, USA).

### 2.2 Isolation of RNA, Synthesis of cDNA, and Analysis of Gene Expression

Total RNA was extracted from the tissues using the modified Trizol method (Rio et al. 2010). The concentration and purity of the isolated RNA were measured with a nucleic acid spectrophotometer (Merinton‐SMA 1000, China). RNA quality was assessed through 1% agarose gel electrophoresis. Prior to cDNA synthesis, genomic DNA contamination was eliminated using DNase I (RNase‐free, Thermo Fisher Scientific, USA, Cat. No: EN0521). cDNA synthesis was performed using a commercial kit (High‐Capacity cDNA Reverse Transcription Kit, Thermo Fisher Scientific, USA, Cat. No: 4368814).

The amplifications of *FABP4*, *C/EBPα*, *SREBP‐1c*, and *PPARγ* genes were determined using Real‐Time PCR (Rotor Gene Q MDx 5plex HRM, Qiagen, USA) with the SYBR Green I kit (Power SYBR Green PCR Master, Thermo Fisher Scientific, USA, Cat. No: 4368702), and each sample was analyzed in triplicate. The qPCR reaction was configured for 40 cycles with 15 s at 95°C and 60 s at 60°C. *RPLP0* and *GAPDH* were used as housekeeping genes, and the primers were designed by the authors of this study using Primer‐BLAST (NCBI) and Primer3Plus (EMBL) (Table 1).

**TABLE 1:** Sequences of primers in the study.

Genes	Forward and reverse sequences	PL (bp)	References
*SREBP‐1c*	F‐5′‐ATCTGTTGGAGCGAGCACTG‐3′ R‐5′‐ATCCGAGGGCATCTGAGAAC‐3′	93	Yakan et al. (2023)
*PPARγ*	F‐5′‐ACTTTGGGATCAGCTCCGTG‐3′ R‐5′‐AACCATCGGGTCAGCTCTTG‐3′	146	Yakan et al. (2023)
*FABP4*	F‐5′‐GGGCCAGGAATTTGATGAAGTC‐3′ R‐5′‐GGTGGTTGATTTCCCATCCCA‐3′	112	^a^
*C/EBPα*	F‐5′‐CATCAGCGCCTACATCGACC‐3′ R‐5′‐CGGGTAGTCAAAGTCGTTGC‐3′	133	^a^
*RPLP0*	F‐5′‐TCCTTCTTCCAGGCTTTAGGC‐3′ R‐5′‐AATCAGCTGCACATCGCTCA‐3′	78	Yakan et al. (2023)
*GAPDH*	F‐5′‐GTCAAGGCAGAGAACGGGAA‐3′ R‐5′‐CCAGCATCACCCCACTTGAT‐3′	95	Yang et al. (2024)

Abbreviations: Bp, base pair; PL, product length.

^a^ Designed in this study.

### 2.3 Western Blotting Application

The protein levels encoded by *FABP4*, *C/EBPα*, *SREBP‐1c*, and *PPARγ* genes were assessed in adipose tissue on the 60th, 90th, and 120th days of feeding. Tissue samples were homogenized using a tissue homogenizer (Biopreb, Allsheng, China) in a solution containing phenylmethylsulfonyl fluoride (PMSF), sodium orthovanadate, a protease inhibitor cocktail, and RIPA buffer (sc‐24948, Santa Cruz, CA, USA). The homogenates were incubated on ice at 4°C for 30 min and then centrifuged at 14,000 ***g*** for 15 min at the same temperature. Following centrifugation, the total protein concentration of the supernatant was measured at 562 nm using an ELISA Reader (AMR‐100; Allsheng, China) with the Pierce BCA Protein Assay Kit (23227, Thermo Fisher Scientific, USA).

For protein separation, 10 μg of total protein from each sample was loaded onto 4%–12% Tris‐Glycine gels. After electrophoresis, the proteins were transferred onto a nitrocellulose membrane (Invitrogen, MA, USA). The membranes were then blocked at room temperature for 1 h with 5% non‐fat dry milk in TBST buffer (10 mM Tris–HCl, pH 7.4, 150 mM NaCl, 0.1% Tween‐20) under gentle shaking. Subsequently, the membranes were incubated overnight at 4°C with primary antibodies diluted in TBST containing 5% non‐fat dry milk.

After incubation with primary antibodies, the membranes were washed three times in TBST and incubated for 1 h at room temperature with secondary antibodies, including goat anti‐rabbit IgG (1:2500, 31460, Thermo Fisher Scientific, USA) and goat anti‐mouse IgG (for GAPDH, 1:10,000, 31430, Thermo Fisher Scientific, USA). The membranes were then washed six times with TBST, and the protein bands were visualized using ECL dye. Band images were captured in a darkroom using the I‐Bright 1500 system (Invitrogen, USA). Quantification of protein signal intensities was performed with ImageJ software (version 1.53k14). GAPDH was used as a housekeeping protein for normalization.

### 2.4 Protein–Protein Interaction (PPI) Network Construction and Module Detection

Differentially expressed genes were mapped to their corresponding proteins and submitted to the STRING database (v12.0; https://string‐db.org/) to retrieve predicted and experimental interactions (Szklarczyk et al. 2025). Only edges with a combined confidence score > 0.7 were retained to ensure high‐confidence associations (Qi et al. 2025). The resulting network was imported into Cytoscape (v3.10.3) for visualization and topological analysis. To identify densely interconnected regions within the PPI network, the MCODE plugin was applied using default parameters (Degree Cutoff = 2; Node Score Cutoff = 0.2; K‐Core = 2; Max. Depth = 100), and the most significant clusters were recorded for downstream functional characterization (Özkan et al. 2025).

### 2.5 Gene Set Enrichment Analysis (GSEA) of Network Proteins

Proteins comprising the PPI network underwent functional enrichment through the Gene Set Enrichment Analysis (GSEA) approach. Gene Ontology categories—Biological Process (GO:BP), Cellular Component (GO:CC), and Molecular Function (GO:MF)—as well as pathway annotations from Kyoto Encyclopedia of Genes and Genomes (KEGG) and WikiPathways were interrogated. From each category, the top ten most enriched terms or pathways were selected and graphically represented using the ggplot2 package (v3.5.1) in R software (v4.4.2).

### 2.6 Statistical Analysis

Statistical analyses were performed using Stata 16.1 statistical software (StataCorp, College Station, TX, USA). Descriptive statistics for each variable were calculated and presented as “Mean ± Standard Error of Mean (SEM)”. The normality of all variables was confirmed using the Shapiro–Wilk test. Fatty acids were analyzed using a linear mixed model. The effect of slaughter day (SLD), PG groups, and their interaction on meat quality parameters and fatty acid composition was examined by using the following model:

$$Y_{\text{ijklm}}=\mu +{\mathrm{SLD}}_{i}+{\mathrm{PG}}_{j}+{\left(\mathrm{SLD}\times \mathrm{PG}\right)}_{\mathrm{ij}}+L_{k}+P_{l}+e_{\text{ijklm}}$$

where *Y*_*ijklm*_ was the observed value for each variable, *μ* was the overall mean, SLD_*i*_ was the effect of the slaughter day (*i* = three classes; Day 60, 90 and 120), PG_*j*_ was the effect of propylene glycol (*j* = three classes; without PG (PG0), PG1.5 and PG3), (SLD × PG)_*ij*_ was the interaction between the slaughter day *i* and propylene glycol *j*, *L*_*k*_ was the random effect of the lambs, *P*_*l*_ was the random effect of pens and *e*_*ijklm*_ was the residual error.

In the model, lambs and pens were assessed as a random effect, while SLD, PG, and their interaction term were assessed as a fixed effect. Variance components were used as the covariance structure in the established model because of resulting in the lowest Akaike information criterion (AIC). When a significant difference was revealed in the model, any significant terms were compared by simple effect analysis with Bonferroni adjustment. While geometric means were determined for housekeeping genes, expression levels of studied genes were calculated with the 2^−∆∆Ct^ method and presented as fold change (Livak and Schmittgen 2001). The effect of PG (PG0, PG1.5, and PG3) on protein expression for each slaughter day was determined using one‐way analysis of variance (ANOVA). To assess the significance of differences between groups regarding the PG effect, Duncan's test was performed for multiple comparisons. *p* < 0.05 was considered significant in all analyses. Statistical significance for Gene Set Enrichment Analysis (GSEA) was assessed via a false discovery rate (FDR) threshold of < 0.01 (Benjamini and Hochberg 1995).

## 3 Results

**FIGURE 1:**
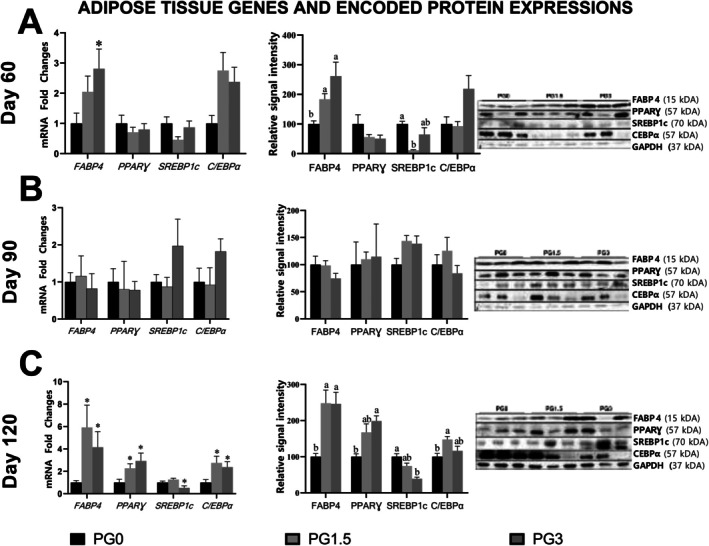
mRNA and protein expression levels of the genes studied in groups.

The analysis of target gene expression levels in gluteal adipose tissue revealed that, on day 60, the expression level of *FABP4* in the PG3 group was approximately 3‐fold upregulated compared to the PG0 group (*p* < 0.05). By day 90, the expression levels of *FABP4*, *C/EBPα*, *PPARγ*, and *SREBP‐1c* showed no significant differences between the groups (*p* > 0.05). However, on day 120, the expression level of *FABP4* was upregulated nearly 6‐fold in the PG1.5 group and more than 4‐fold in the PG3 group compared to the PG0 group (*p* < 0.05). Similarly, the expression levels of *C/EBPα* and *PPARγ* were approximately 3‐fold upregulated in the PG1.5 and PG3 groups compared to the PG0 group (*p* < 0.05). In contrast, the expression level of *SREBP‐1c* was significantly downregulated, showing an approximately 2‐fold decrease in the PG3 group compared to the PG0 group (*p* < 0.05) (Figure 1A). On day 60, the expression level of the FABP4 protein was found to be significantly increased in the PG1.5 and PG3 groups compared to the PG0 group (*p* < 0.01). Conversely, the expression level of the SREBP‐1c protein was approximately 10‐fold downregulated in the PG1.5 group compared to the PG0 group (*p* < 0.05). Additionally, changes in the expression levels of C/EBPα and PPARγ proteins in gluteal adipose tissue on day 60 were not significant among the groups (Figure 1A). On day 90, no significant changes were observed in the expression levels of *FABP4*, *C/EBPα*, *PPARγ*, and *SREBP‐1c* proteins in gluteal adipose tissue (*p* > 0.05). The findings were presented in Figure 1B. On day 120, the expression level of the FABP4 protein in gluteal adipose tissue was approximately 2.5‐fold higher in the PG1.5 and PG3 groups compared to the PG0 group (*p* < 0.01). Similarly, for PPARγ protein, an increase was observed in both the PG1.5 and PG3 groups relative to the PG0 group; however, the increase was significant only in the PG3 group (*p* < 0.01). In terms of C/EBPα protein expression, a significant increase was observed in the PG1.5 group compared to the PG0 group (*p* < 0.05), while the expression level of the SREBP‐1c protein was significantly decreased in the PG3 group relative to the PG0 group (*p* < 0.001) (Figure 1C).

The mean values of fatty acid composition for the experimental groups and different slaughter times are presented in Table 2. While all fatty acids, except for C20:0, were similar across the experimental groups, significant differences were observed for C12:0, C14:0, C15:0, C17:0, C17:1, C18:3 n3, C22:0, C22:6 n3, C24:0, and C24:1 with respect to slaughter time (*p* < 0.05, *p* < 0.01, and *p* < 0.001). For the most abundant fatty acids, including C16:0, C16:1, C18:0, and C18:1, similar values were observed for both the experimental groups and slaughter times.

**TABLE 2:** Fatty acid composition in the adipose tissue for different experimental groups and slaughter days (Mean ± SE).

Fatty acids (%)	Group (G)	Slaughter day (SLD)			Mean (G)	*p*		
		Day 60	Day 90	Day 120		G	SLD	GxSLD
C8:0	PG0	0.03 ± 0.02	0.02 ± 0.004	0.02 ± 0.003	0.03 ± 0.01	—	—	—
	PG1.5	0.04 ± 0.02	0.02 ± 0.002	0.02 ± 0.002	0.03 ± 0.01			
	PG3	0.02 ± 0.001	0.02 ± 0.001	0.03 ± 0.02	0.02 ± 0.01			
	Mean (SLD)	0.03 ± 0.01	0.02 ± 0.002	0.02 ± 0.01				
C10:0	PG0	0.41 ± 0.07	0.52 ± 0.11	0.34 ± 0.02	0.42 ± 0.04	—	—	—
	PG1.5	0.50 ± 0.05	0.37 ± 0.05	0.33 ± 0.01	0.41 ± 0.03			
	PG3	0.35 ± 0.03	0.39 ± 0.04	0.31 ± 0.03	0.35 ± 0.02			
	Mean (SLD)	0.42 ± 0.03	0.42 ± 0.04	0.33 ± 0.01				
C11:0	PG0	0.10 ± 0.05	0.06 ± 0.01	0.06 ± 0.005	0.07 ± 0.02	—	—	—
	PG1.5	0.05 ± 0.01	0.12 ± 0.07	0.07 ± 0.004	0.08 ± 0.02			
	PG3	0.05 ± 0.01	0.05 ± 0.003	0.06 ± 0.01	0.05 ± 0.004			
	Mean (SLD)	0.07 ± 0.02	0.08 ± 0.02	0.06 ± 0.005				
C12:0	PG0	0.53 ± 0.10	0.43 ± 0.08	0.26 ± 0.01	0.42 ± 0.05	—	***	—
	PG1.5	0.62 ± 0.12	0.31 ± 0.04	0.22 ± 0.02	0.41 ± 0.06			
	PG3	0.37 ± 0.03	0.38 ± 0.05	0.26 ± 0.04	0.34 ± 0.03			
	Mean (SLD)	0.51 ± 0.06^a^	0.37 ± 0.03^ab^	0.25 ± 0.02^b^				
C13:0	PG0	0.10 ± 0.03	0.09 ± 0.02	0.09 ± 0.02	0.09 ± 0.01	—	—	—
	PG1.5	0.08 ± 0.01	0.10 ± 0.01	0.11 ± 0.01	0.09 ± 0.01			
	PG3	0.08 ± 0.01	0.07 ± 0.01	0.12 ± 0.02	0.09 ± 0.01			
	Mean (SLD)	0.09 ± 0.01	0.08 ± 0.01	0.11 ± 0.01				
C14:0	PG0	8.21 ± 0.53	6.59 ± 0.35	6.99 ± 0.20	7.36 ± 0.28	—	**	—
	PG1.5	8.21 ± 0.72	6.48 ± 0.31	5.68 ± 0.45	6.97 ± 0.40			
	PG3	6.54 ± 0.35	6.69 ± 0.43	6.44 ± 0.23	6.55 ± 0.19			
	Mean (SLD)	7.70 ± 0.35^a^	6.59 ± 0.21^b^	6.41 ± 0.19^b^				
C14:1	PG0	0.68 ± 0.04	0.83 ± 0.06	0.84 ± 0.04	0.78 ± 0.03	—	—	—
	PG1.5	0.90 ± 0.13	0.85 ± 0.07	0.91 ± 0.06	0.89 ± 0.06			
	PG3	0.88 ± 0.06	0.80 ± 0.05	0.89 ± 0.07	0.85 ± 0.04			
	Mean (SLD)	0.82 ± 0.05	0.83 ± 0.03	0.88 ± 0.04				
C15:0	PG0	1.92 ± 0.26	2.27 ± 0.18	2.44 ± 0.17	2.18 ± 0.13	—	***	—
	PG1.5	1.88 ± 0.21	2.47 ± 0.24	3.15 ± 0.18	2.41 ± 0.17			
	PG3	2.18 ± 0.24	1.96 ± 0.13	2.67 ± 0.25	2.27 ± 0.13			
	Mean (SLD)	1.99 ± 0.13^b^	2.22 ± 0.11^b^	2.72 ± 0.14^a^				
C15:1	PG0	0.75 ± 0.10	0.97 ± 0.14	0.94 ± 0.08	0.87 ± 0.06	—	—	—
	PG1.5	0.76 ± 0.16	1.14 ± 0.19	0.94 ± 0.21	0.94 ± 0.11			
	PG3	1.07 ± 0.14	0.89 ± 0.10	0.94 ± 0.15	0.96 ± 0.07			
	Mean (SLD)	0.85 ± 0.08	1.00 ± 0.08	0.94 ± 0.08				
C16:0	PG0	28.70 ± 0.45	26.88 ± 1.98	28.60 ± 0.51	28.12 ± 0.63	—	—	—
	PG1.5	26.52 ± 1.79	29.24 ± 0.74	26.23 ± 1.60	27.40 ± 0.87			
	PG3	28.16 ± 0.65	29.47 ± 0.25	28.26 ± 0.59	28.65 ± 0.31			
	Mean (SLD)	27.78 ± 0.67	28.65 ± 0.64	27.83 ± 0.53				
C16:1	PG0	4.50 ± 0.36	5.09 ± 0.50	5.08 ± 0.25	4.85 ± 0.22	—	—	—
	PG1.5	5.20 ± 0.53	4.66 ± 0.47	5.73 ± 0.48	5.14 ± 0.30			
	PG3	5.00 ± 0.33	4.27 ± 0.27	5.04 ± 0.42	4.76 ± 0.20			
	Mean (SLD)	4.89 ± 0.24	4.63 ± 0.23	5.24 ± 0.23				
C17:0	PG0	2.27 ± 0.18	2.29 ± 0.39	2.79 ± 0.14	2.43 ± 0.15	—	*	—
	PG1.5	2.19 ± 0.30	2.83 ± 0.24	3.37 ± 0.10	2.71 ± 0.18			
	PG3	2.65 ± 0.26	2.61 ± 0.17	2.62 ± 0.23	2.63 ± 012			
	Mean (SLD)	2.36 ± 0.15^b^	2.59 ± 0.15^ab^	2.87 ± 0.13^a^				
C17:1	PG0	1.97 ± 0.22	2.27 ± 0.20	2.30 ± 0.20	2.16 ± 0.12	—	*	—
	PG1.5	2.08 ± 0.28	2.46 ± 0.27	3.17 ± 0.20	2.48 ± 0.18			
	PG3	2.33 ± 0.23	2.11 ± 0.16	2.37 ± 0.20	2.27 ± 0.11			
	Mean (SLD)	2.12 ± 0.14^b^	2.27 ± 0.12^ab^	2.56 ± 0.14^a^				
C18:0	PG0	14.77 ± 0.72	15.72 ± 0.81	14.69 ± 0.92	15.03 ± 0.45	—	—	—
	PG1.5	13.54 ± 0.93	13.63 ± 1.13	13.30 ± 1.03	13.51 ± 0.57			
	PG3	14.49 ± 0.90	15.11 ± 0.65	14.59 ± 0.74	14.74 ± 0.42			
	Mean (SLD)	14.26 ± 0.48	14.79 ± 0.52	14.28 ± 0.49				
C18:1	PG0	30.72 ± 0.56	31.67 ± 0.99	30.32 ± 0.80	30.89 ± 0.44	—	—	—
	PG1.5	33.05 ± 0.73	30.94 ± 0.60	32.27 ± 0.62	32.12 ± 0.43			
	PG3	31.24 ± 0.66	30.97 ± 0.26	31.43 ± 0.53	31.21 ± 0.28			
	Mean (SLD)	31.69 ± 0.42	31.16 ± 0.35	31.30 ± 0.40				
C18:2 n6	PG0	2.72 ± 0.14	2.95 ± 0.32	2.78 ± 0.14	2.81 ± 0.11	—	—	—
	PG1.5	2.91 ± 0.14	2.94 ± 0.13	3.08 ± 0.39	2.96 ± 0.11			
	PG3	3.03 ± 0.13	2.76 ± 0.13	2.63 ± 0.08	2.80 ± 0.07			
	Mean (SLD)	2.88 ± 0.08	2.88 ± 0.11	2.80 ± 0.12				
C20:0	PG0	0.06 ± 0.01	0.07 ± 0.02	0.06 ± 0.01	0.06 ± 0.01^A^	*	—	—
	PG1.5	0.05 ± 0.003	0.05 ± 0.004	0.05 ± 0.003	0.05 ± 0.002^B^			
	PG3	0.06 ± 0.004	0.05 ± 0.003	0.05 ± 0.002	0.05 ± 0.002^B^			
	Mean (SLD)	0.06 ± 0.003	0.06 ± 0.01	0.05 ± 0.003				
C18:3 n3	PG0	0.55 ± 0.06	0.51 ± 0.07	0.40 ± 0.03	0.49 ± 0.03	—	***	—
	PG1.5	0.57 ± 0.04	0.44 ± 0.02	0.42 ± 0.02	0.49 ± 0.02			
	PG3	0.54 ± 0.04	0.47 ± 0.01	0.40 ± 0.03	0.47 ± 0.02			
	Mean (SLD)	0.56 ± 0.03^a^	0.47 ± 0.02^b^	0.41 ± 0.02^b^				
C20:1	PG0	0.78 ± 0.07	0.55 ± 0.15	0.84 ± 0.03	0.73 ± 0.06	—	—	—
	PG1.5	0.68 ± 0.05	0.79 ± 0.05	0.78 ± 0.04	0.74 ± 0.03			
	PG3	0.76 ± 0.06	0.70 ± 0.04	0.72 ± 0.05	0.73 ± 0.03			
	Mean (SLD)	0.74 ± 0.03	0.69 ± 0.05	0.77 ± 0.03				
C18:3 n6	PG0	0.08 ± 0.06	0.03 ± 0.01	0.01 ± 0.001	0.04 ± 0.02	—	—	—
	PG1.5	0.02 ± 0.01	0.01 ± 0.001	0.02 ± 0.01	0.01 ± 0.003			
	PG3	0.02 ± 0.004	0.08 ± 0.06	0.02 ± 0.01	0.04 ± 0.02			
	Mean (SLD)	0.04 ± 0.02	0.04 ± 0.02	0.01 ± 0.003				
C20:2 n6	PG0	0.03 ± 0.002	0.03 ± 0.002	0.03 ± 0.0008	0.03 ± 0.0009	—	—	—
	PG1.5	0.03 ± 0.001	0.03 ± 0.003	0.03 ± 0.002	0.03 ± 0.001			
	PG3	0.03 ± 0.001	0.03 ± 0.001	0.03 ± 0.008	0.03 ± 0.0006			
	Mean (SLD)	0.03 ± 0.0008	0.03 ± 0.001	0.03 ± 0.007				
C22:0	PG0	0.02 ± 0.001	0.02 ± 0.004	0.02 ± 0.002	0.02 ± 0.001	—	***	—
	PG1.5	0.03 ± 0.001	0.02 ± 0.001	0.02 ± 0.001	0.02 ± 0.001			
	PG3	0.02 ± 0.002	0.02 ± 0.0006	0.02 ± 0.001	0.02 ± 0.0009			
	Mean (SLD)	0.03 ± 0.0009^a^	0.02 ± 0.001^b^	0.02 ± 0.0009^b^				
C20:3 n6	PG0	0.01 ± 0.001	0.01 ± 0.002	0.01 ± 0.0003	0.01 ± 0.0008	—	—	—
	PG1.5	0.01 ± 0.0007	0.01 ± 0.0005	0.01 ± 0.01	0.01 ± 0.002			
	PG3	0.01 ± 0.005	0.01 ± 0.0004	0.01 ± 0.0002	0.01 ± 0.0002			
	Mean (SLD)	0.01 ± 0.0005	0.01 ± 0.0006	0.01 ± 0.002				
C22:1	PG0	0.01 ± 0.001	0.01 ± 0.002	0.01 ± 0.0008	0.01 ± 0.0001	—	—	—
	PG1.5	0.01 ± 0.001	0.01 ± 0.001	0.01 ± 0.001	0.01 ± 0.0006			
	PG3	0.01 ± 0.002	0.01 ± 0.0005	0.01 ± 0.0005	0.01 ± 0.0006			
	Mean (SLD)	0.01 ± 0.0008	0.01 ± 0.0008	0.01 ± 0.0004				
C20:3 n3	PG0	0.01 ± 0.0007	0.01 ± 0.001	0.01 ± 0.001	0.01 ± 0.0006	—	—	—
	PG1.5	0.01 ± 0.0009	0.01 ± 0.002	0.01 ± 0.0007	0.01 ± 0.0007			
	PG3	0.01 ± 0.002	0.01 ± 0.0009	0.01 ± 0.0009	0.01 ± 0.0006			
	Mean (SLD)	0.01 ± 0.0006	0.01 ± 0.0008	0.01 ± 0.0005				
C20:4 n6	PG0	0.05 ± 0.003	0.05 ± 0.005	0.04 ± 0.003	0.05 ± 0.002	—	—	—
	PG1.5	0.05 ± 0.01	0.04 ± 0.004	0.04 ± 0.005	0.04 ± 0.003			
	PG3	0.05 ± 0.01	0.04 ± 0.002	0.04 ± 0.003	0.05 ± 0.002			
	Mean (SLD)	0.05 ± 0.003	0.04 ± 0.002	0.04 ± 0.002				
C22:2 n6	PG0	0.003 ± 0.0003	0.003 ± 0.0006	0.002 ± 0.00009	0.002 ± 0.0002	—	—	—
	PG1.5	0.003 ± 0.0003	0.002 ± 0.0002	0.002 ± 0.0004	0.002 ± 0.0001			
	PG3	0.002 ± 0.0002	0.002 ± 0.0001	0.002 ± 0.0002	0.002 ± 0.0001			
	Mean (SLD)	0.003 ± 0.0002	0.002 ± 0.0002	0.002 ± 0.0001				
C24:0	PG0	0.008 ± 0.001	0.006 ± 0.0008	0.004 ± 0.0005	0.006 ± 0.0006	—	***	—
	PG1.5	0.01 ± 0.002	0.006 ± 0.0006	0.005 ± 0.002	0.008 ± 0.001			
	PG3	0.009 ± 0.001	0.006 ± 0.0006	0.005 ± 0.0005	0.007 ± 0.0005			
	Mean (SLD)	0.009 ± 0.0009^a^	0.006 ± 0.0004^b^	0.005 ± 0.0005^b^				
C20:5 n3	PG0	0.007 ± 0.0008	0.009 ± 0.001	0.009 ± 0.0009	0.008 ± 0.0005	—	—	—
	PG1.5	0.008 ± 0.0006	0.01 ± 0.001	0.009 ± 0.001	0.009 ± 0.0005			
	PG3	0.008 ± 0.0006	0.007 ± 0.0008	0.008 ± 0.0006	0.008 ± 0.0004			
	Mean (SLD)	0.008 ± 0.0004	0.008 ± 0.0006	0.008 ± 0.0005				
C24:1	PG0	0.02 ± 0.002	0.02 ± 0.001	0.01 ± 0.0009	0.02 ± 0.001	—	***	—
	PG1.5	0.03 ± 0.002	0.02 ± 0.002	0.01 ± 0.002	0.02 ± 0.002			
	PG3	0.02 ± 0.002	0.02 ± 0.001	0.01 ± 0.002	0.02 ± 0.001			
	Mean (SLD)	0.02 ± 0.002 ^a^	0.02 ± 0.0009^a^	0.01 ± 0.0008^b^				
C22:6 n3	PG0	0.008 ± 0.0009	0.007 ± 0.0008	0.007 ± 0.0005	0.007 ± 0.0005	—	**	—
	PG1.5	0.009 ± 0.001	0.007 ± 0.0003	0.006 ± 0.0004	0.008 ± 0.0006			
	PG3	0.01 ± 0.0007	0.008 ± 0.0005	0.007 ± 0.0005	0.008 ± 0.0004			
	Mean (SLD)	0.009 ± 0.0006^a^	0.007 ± 0.0003^b^	0.007 ± 0.0003^b^				

*Note:* —*p* > 0.05; *p < 0.05; **p < 0.01; ***p < 0.001. ^a,b^Differences between different letters in the same row are significant (*p* < 0.05).^A,B^Differences between different letters in the same column are significant (*p* < 0.05).

The total and index values derived from the fatty acid composition were presented in Table 3. In the PG1.5 group, the SFA value was lower compared to the other groups, while the MUFA and UFA values were higher (*p* < 0.05). Additionally, the lower AI and TI values in the PG1.5 group were identified as significant findings (*p* < 0.05). Regarding the slaughter time evaluation, it was determined that the total n3 content decreased as the slaughter day progressed (*p* < 0.001), while the n6/n3 ratio increased (*p* < 0.01).

**TABLE 3:** Means of the total and index values obtained from the fatty acid composition in the adipose tissue for different experimental groups and slaughter days (Mean ± SE).

Parameters (%)	Group (G)	Slaughter day (SLD)			Mean (G)	*p*		
		Day 60	Day 90	Day 120		G	SLD	GxSLD
SFA	PG0	57.13 ± 0.73	54.98 ± 1.67	56.36 ± 0.97	56.26 ± 0.65^A^	*	—	—
	PG1.5	53.72 ± 1.16	55.64 ± 1.15	52.55 ± 1.23	54.10 ± 0.71^B^			
	PG3	54.99 ± 0.84	56.81 ± 0.34	55.43 ± 0.57	55.77 ± 0.37^AB^			
	Mean (SLD)	55.29 ± 0.60	55.89 ± 0.61	54.96 ± 0.59				
MUFA	PG0	39.45 ± 0.74	41.42 ± 1.42	40.34 ± 0.99	40.31 ± 0.59^B^	*	—	—
	PG1.5	42.71 ± 1.08	40.86 ± 1.22	43.83 ± 0.88	42.35 ± 0.67^A^			
	PG3	41.30 ± 0.81	39.77 ± 0.35	41.42 ± 0.52	40.81 ± 0.35^AB^			
	Mean (SLD)	41.15 ± 0.57	40.61 ± 0.58	41.72 ± 0.52				
PUFA	PG0	3.47 ± 0.20	3.60 ± 0.36	3.29 ± 0.17	3.45 ± 0.14	—	—	—
	PG1.5	3.62 ± 0.15	3.50 ± 0.14	3.62 ± 0.43	3.58 ± 0.12			
	PG3	3.71 ± 0.17	3.42 ± 0.15	3.15 ± 0.09	3.42 ± 0.09			
	Mean (SLD)	3.59 ± 0.10	3.50 ± 0.12	3.32 ± 0.13				
UFA	PG0	42.92 ± 0.72	45.02 ± 1.67	43.64 ± 0.97	43.76 ± 0.64^B^	*	—	—
	PG1.5	46.33 ± 1.16	44.36 ± 1.15	47.46 ± 1.23	45.92 ± 0.71^A^			
	PG3	45.01 ± 0.84	43.19 ± 0.34	44.57 ± 0.57	44.23 ± 0.37^AB^			
	Mean (SLD)	44.74 ± 0.60	44.11 ± 0.61	45.04 ± 0.59				
PUFA/SFA	PG0	0.06 ± 0.004	0.07 ± 0.01	0.06 ± 0.003	0.06 ± 0.003	—	—	—
	PG1.5	0.07 ± 0.004	0.06 ± 0.002	0.07 ± 0.01	0.07 ± 0.003			
	PG3	0.07 ± 0.003	0.06 ± 0.003	0.06 ± 0.002	0.06 ± 0.002			
	Mean (SLD)	0.07 ± 0.002	0.06 ± 0.003	0.06 ± 0.003				
UFA/SFA	PG0	0.75 ± 0.02	0.83 ± 0.06	0.78 ± 0.03	0.78 ± 0.02^B^	*	—	—
	PG1.5	0.87 ± 0.04	0.80 ± 0.04	0.91 ± 0.05	0.86 ± 0.03^A^			
	PG3	0.82 ± 0.03	0.76 ± 0.01	0.81 ± 0.02	0.79 ± 0.01^B^			
	Mean (SLD)	0.81 ± 0.02	0.79 ± 0.02	0.82 ± 0.02				
n6	PG0	2.89 ± 0.19	3.07 ± 0.33	2.87 ± 0.14	2.94 ± 0.13	—	—	—
	PG1.5	3.02 ± 0.14	3.03 ± 0.13	3.18 ± 0.41	3.06 ± 0.12			
	PG3	3.14 ± 0.13	2.93 ± 0.14	2.73 ± 0.08	2.92 ± 0.08			
	Mean (SLD)	3.01 ± 0.09	3.00 ± 0.11	2.89 ± 0.12				
n3	PG0	0.57 ± 0.06	0.53 ± 0.07	0.43 ± 0.03	0.52 ± 0.03	—	***	—
	PG1.5	0.60 ± 0.04	0.47 ± 0.02	0.44 ± 0.02	0.51 ± 0.02			
	PG3	0.57 ± 0.05	0.50 ± 0.02	0.43 ± 0.02	0.49 ± 0.02			
	Mean (SLD)	0.58 ± 0.03^a^	0.50 ± 0.02^b^	0.43 ± 0.02^b^				
n6/n3	PG0	5.37 ± 0.58	5.97 ± 0.67	6.87 ± 0.42	6.00 ± 0.35	—	**	—
	PG1.5	5.16 ± 0.41	6.52 ± 0.31	7.18 ± 0.55	6.14 ± 0.30			
	PG3	5.67 ± 0.33	5.89 ± 0.17	6.54 ± 0.38	6.05 ± 0.19			
	Mean (SLD)	5.39 ± 0.26^b^	6.12 ± 0.22^ab^	6.81 ± 0.25^a^				
NV	PG0	1.59 ± 0.05	1.84 ± 0.22	1.58 ± 0.04	1.66 ± 0.07	—	—	—
	PG1.5	1.85 ± 0.20	1.53 ± 0.06	1.78 ± 0.19	1.72 ± 0.10			
	PG3	1.63 ± 0.07	1.56 ± 0.03	1.64 ± 0.06	1.61 ± 0.03			
	Mean (SLD)	1.69 ± 0.08	1.63 ± 0.07	1.66 ± 0.06				
AI	PG0	1.13 ± 0.06	0.95 ± 0.06	0.99 ± 0.04	1.03 ± 0.04^A^	*	*	—
	PG1.5	1.02 ± 0.07	0.91 ± 0.05	0.77 ± 0.04	0.92 ± 0.04^B^			
	PG3	0.92 ± 0.05	0.98 ± 0.03	0.91 ± 0.03	0.94 ± 0.02^B^			
	Mean (SLD)	1.03 ± 0.04^a^	0.95 ± 0.03^ab^	0.90 ± 0.03^b^				
TI	PG0	1.35 ± 0.04	1.25 ± 0.09	1.32 ± 0.06	1.31 ± 0.04^A^	*	—	—
	PG1.5	1.17 ± 0.06	1.27 ± 0.06	1.10 ± 0.05	1.19 ± 0.04^B^			
	PG3	1.24 ± 0.04	1.33 ± 0.02	1.26 ± 0.03	1.28 ± 0.02^AB^			
	Mean (SLD)	1.26 ± 0.03	1.29 ± 0.03	1.24 ± 0.03				

*Note:* —*p* > 0.05; *p < 0.05; **p < 0.01; ***p < 0.001. ^a,b^The differences between different letters within the same row are statistically significant (*p* < 0.05). ^A,B^The differences between different letters within the same column are statistically significant (*p* < 0.05). SFA (Saturated fatty acids) = (C8:0 + C10:0 + C11:0 + C12:0 + C13:0 + C14:0 + C15:0 + C16:0 + C17:0 + C18:0 + C20:0 + C22:0 + C24:0). MUFA (Monounsaturated fatty acids) = (C14:1 + C15:1 + C16:1 + C17:1 + C18:1 + C20:1 + C22:1 + C24:1). n6 (Omega 6) = (C18:2 n6 + C18:3 n6 + C20:2 n6 + C20:3 n6 + C20:4 n6 + C22:2 n6). n3 (Omega 3) = (C18:3 n3 + C20:3 n3 + C20:5 n3 + C22:6 n3). PUFA (Polyunsaturated fatty acids) = n6 + n3; UFA (Unsaturated fatty acids) = MUFA + PUFA. TI (Thrombogenic index) = (C14:0 + C16:0 + C18:0)/(0.5*C18:1) + (0.5*ΣMUFA) + (0.5*Σn6) + (3*Σn3) + (Σn3/Σn6). AI (Atherogenic index) = (C12:0 + (4*C14:0) + C18:0)/ΣUFA; NV (Nutritional value) = (C18:0 + C18:1)/C16:0.

**FIGURE 2:**
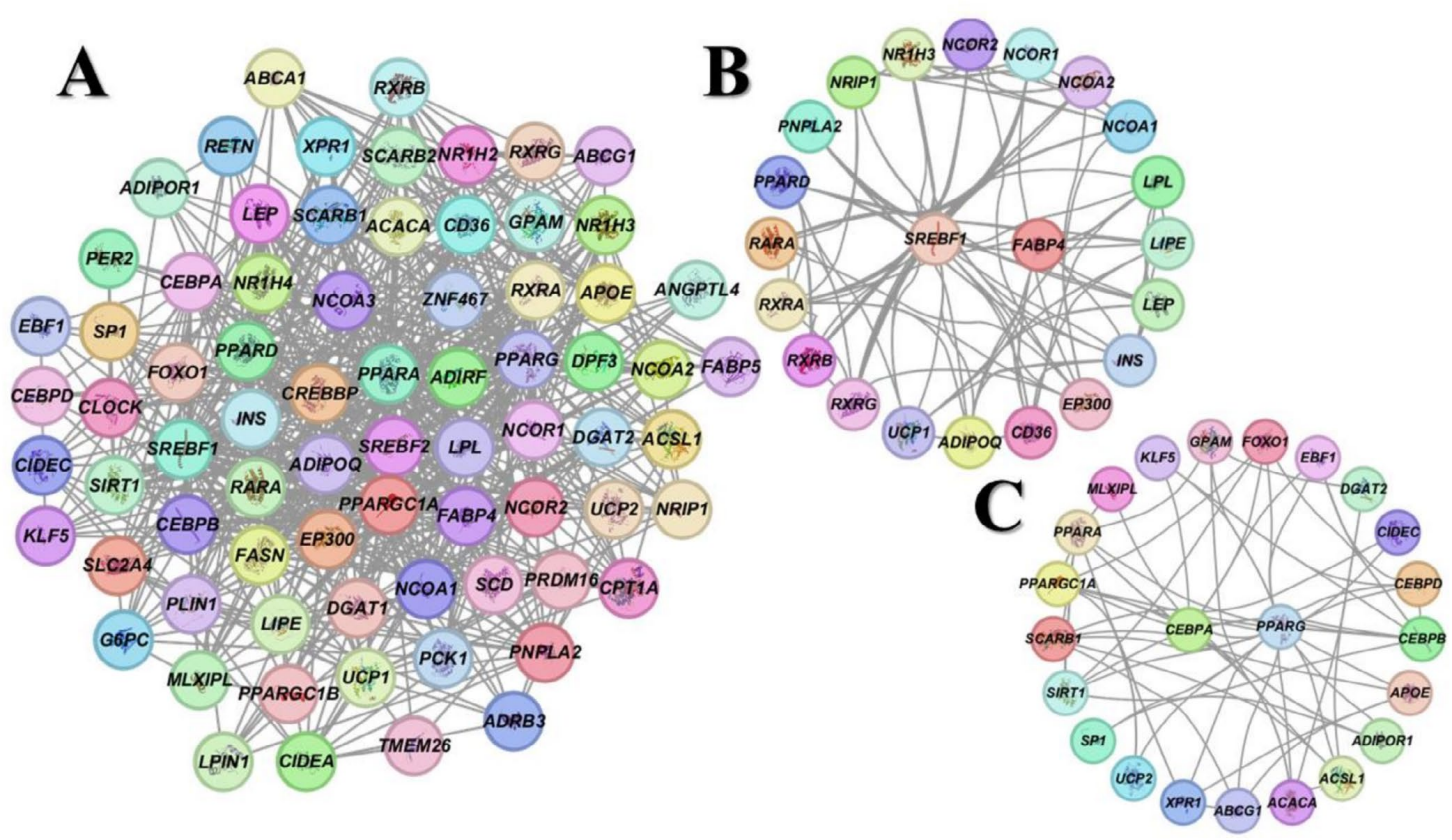
(A) Global PPI network of 74 proteins (615 edges); (B) MCODE Cluster 1 (score 10.571; 23 nodes, 65 edges); (C) MCODE Cluster 2 (score 5.909; 22 nodes, 111 edges).

Protein–protein interaction (PPI) network analysis revealed that 74 proteins, including FABP4, PPARγ, SREBP‐1c, and CEBPα, were interconnected via 615 edges (Figure 2). Using the MCODE plugin in Cytoscape, two highly dense modules were identified: Module 1 (MCODE score 10.571) comprised 23 proteins and 65 edges, and Module 2 (MCODE score 5.909) comprised 22 proteins and 111 edges. In Module 1, SREBP‐1c and FABP4 acted as seed nodes, and the cluster included fatty acid metabolism regulators such as RXRA, RXRB, RXRG, leptin (LEP), and adiponectin (ADIPOQ). Module 2 centered on core regulators of lipid metabolism and cellular homeostasis, namely SIRT1, FOXO1, and PPARα, forming a tightly interconnected subnetwork.

**FIGURE 3:**
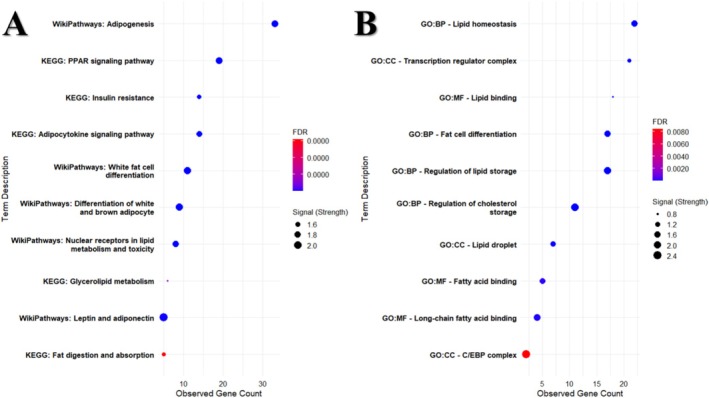
(A) Signal pathway enrichment from KEGG and WikiPathways databases; (B) Gene Ontology enrichment for the network proteins, BP (Biological Process); CC (Cellular Component); MF (Molecular Function).

To elucidate the roles of highly interconnected proteins in organismal signaling and metabolic processes, we performed gene set enrichment analysis (Figure 3). Our analysis revealed that more than 20 of the target genes were significantly enriched in adipogenesis and PPAR signaling pathways (FDR < 0.01), highlighting their central role in lipid accumulation and transcriptional control of fat cell differentiation. Moreover, these genes showed significant associations with insulin resistance, energy metabolism, and the regulation of adipocyte maturation. Subsequent Gene Ontology (GO) enrichment pinpointed these proteins as key regulators of lipid homeostasis and lipid metabolic processes. Specifically, GO terms related to lipid storage and cholesterol handling were overrepresented, indicating that these factors collectively orchestrate the formation and maintenance of adipose tissue lipid droplets.

## 4 Discussion

When analyzing fatty acid composition, it was determined that C16:0, C18:0, and C18:1 had the highest proportions across all slaughter days and groups. These three fatty acids accounted for more than 70% of the total fatty acids. In experimental groups, PG had no effect except for C20:0, suggesting that PG does not individually influence the fatty acids stored as depot fat. Fatty acid synthesis occurs within mitochondria, starting from four‐carbon precursors and proceeding through a series of biochemical elongation reactions involving malonyl and acetyl groups. Once the 16‐carbon saturated palmitate (C16:0) is formed, chain elongation ceases. Following this stage, the desaturation of C16:0 begins, with the primary enzyme responsible for this process being stearoyl‐CoA desaturase (SCD). Two essential molecules required for these processes are acetyl‐CoA and glucose (Murray et al. 2009). It was also reported by Yakan et al. (2024) that PG has an effect on C20:0 in these processes. C20:0 decreased in the PG0 group due to PG utilization (*p* < 0.05). This can be considered an advantage of PG use.

When PG is administered orally, it reaches the liver via the rumen‐hepatic cycle and is directly utilized in glucose production (Studer et al. 1993; Trabue et al. 2007). Therefore, the increased gluconeogenesis in the liver due to PG supplementation is thought to lead to a reduction in short‐chain saturated fatty acids (SCFA) being converted into C16:0 in depot fat, and subsequently, the desaturation of these fatty acids results in a decreased proportion of de novo synthesized saturated fatty acids (SFA) while increasing unsaturated fatty acid (UFA) levels. In the present study, the observed reduction in SFA levels (*p* < 0.05) and the increase in UFA levels (*p* < 0.05) in the GFTs group following PG supplementation can be explained by this mechanism. Furthermore, AI and TI values for GFTs were found to decrease with PG supplementation (*p* < 0.05), which are considered a significant advantage in terms of human nutrition.

It has been observed that the levels of certain individual fatty acids C12:0, C14:0, C18:3n3, C22:0, C24:0, C24:1, and C22:6n3 in GFTs decrease with increasing slaughter age. Two important points have been identified in this observation. First, as a result of the decrease in n3 fatty acids (C18:3n3 and C22:6n3), there is an increase in the n6/n3 ratio. This is considered a negative situation in human nutrition according to the (Department of Health 1994). It is also reported in other studies that slaughtering lambs at low live weight will be favorable in terms of human health as the live weight will increase due to the increase in slaughter day (D'Alessandro et al. 2015; Yakan et al. 2024). The second important point is that C12:0 and C14:0 fatty acids, which have an important place in the amount of individual fatty acids, decrease with the increase in slaughter weight. This is consistent with the report of Ekiz et al. (2019). While the change determined by Ekiz et al. (2019) was reported in the intramuscular fat of *Musculus longissimus dorsi*, it is understood that a similar change was also observed in GFTs, which is storage fat. On the other hand, the change in these fatty acids causes a significant change in the SFA ratio.

The FABP protein family actively regulates intracellular fatty acid levels by directly influencing anabolic and catabolic reactions (Masarwi et al. 2019). Additionally, this family modulates the activity of genes involved in fatty acid regulation (Wang et al. 2019). *FABP4*, highly expressed in adipose tissue, plays a critical role in glucose homeostasis, fatty acid transport, absorption, and metabolism (Smathers and Petersen 2011; Pewan et al. 2020; Ye et al. 2022). Studies have shown that changes in *FABP4* gene expression can serve as a biomarker for feed efficiency in cattle and sheep (Cohen‐Zinder et al. 2019; Arce‐Recinos et al. 2022). Han et al. (2021) reported that sheep breeds with fat tails exhibit higher FABP4 mRNA and protein activity compared to lean‐tailed breeds, suggesting a relationship with tail fat content (Han et al. 2021). Yang, Ahmad, et al. (2020), Yang, Wang, et al. (2020) observed a significant increase in subcutaneous adipose tissue *FABP4* gene expression after feeding yaks a high‐energy diet for 60 days. Similarly, in the current study, FABP4 mRNA and protein levels increased on the 60th day, depending on dietary energy content. While *FABP4* mRNA expression was significantly upregulated only in the PG3 group compared to the PG0, FABP4 protein levels were significantly higher in both the PG1.5 and PG3 groups than in the PG0. This suggests that fatty acid metabolism in sheep may be regulated similarly to yaks at the molecular levels.

The current study found that adipose tissue FABP4 mRNA expression and protein levels significantly increased in 120‐day slaughter samples compared to the PG0 group. This increase may be associated with a reduction in growth rates due to feeding duration and increased carcass fat accumulation in lambs (Bakhtiarizadeh et al. 2013; Han et al. 2021). *FABP4* gene expression levels in adipose tissue were significantly downregulated in the PG0, PG1.5, and PG3 groups on the 90th and 120th days compared to the 60th day. This time‐dependent decrease in *FABP4* gene expression might be related to an increase in adipocytes during adipose tissue growth (Zhang et al. 2008). Other studies have indicated that adipose tissue development, regulated by hormones such as leptin during the growth process, particularly influences genes like FABP4 (Nguyen‐Tu et al. 2021; Gan et al. 2015). This study on Akkaraman lambs showed that FABP4 activity in adipose tissue decreased over time, independent of diet content, as growth progressed.

The adipogenic transcription factor PPARγ upregulates genes like FABP4 involved in energy metabolism, playing a key role in processes such as adipogenesis, lipid metabolism, and insulin sensitivity (Wang et al. 2020). In adipose tissue, upregulation of PPARγ facilitates storage of dietary energy as fatty acids and triacylglycerols (Fan et al. 2019), while its downregulation reduces adipokine secretion, leading to adipose tissue atrophy (González et al. 2019). Studies have reported that *PPARγ* gene expression promotes adipocyte differentiation (Sun et al. 2021). In cattle, PPARγ is highly expressed in adipose tissue and is influenced by dietary energy levels, regulating lipogenesis (Minuti et al. 2020). In sheep, high‐energy prenatal diets have been shown to increase offspring *PPARγ* gene expression, whereas low‐energy diets decrease it (Khanal et al. 2020). However, studies by Coleman et al. (2019), Mirzaei‐Alamouti et al. (2021) found no significant changes in *PPARγ* mRNA expression in sheep adipose tissue following high‐energy diets supplemented with eicosapentaenoic acid, docosahexaenoic acid, sunflower oil, or fish oil. In the current study, 60‐ and 90‐day PG supplementation did not significantly affect PPARγ gene expression or protein levels in adipose tissue. However, on the 120th day, *PPARγ* gene expression levels increased in both the PG1.5 and PG3 groups, while protein levels were significantly higher only in the PG3 group. This suggests that the increase in PPARγ mRNA and protein levels in the 120‐day PG3 group might be related to the dietary energy content and the role of PG in promoting fat accumulation as growth slows (Fan et al. 2019). Further findings revealed that adipose tissue PPARγ activity decreased in all groups as feeding progressed from the 60th to the 120th day. This decline in gene activity may be associated with reduced adipogenesis and growth‐related changes in adipose tissue development (Lee et al. 2020; Kim et al. 2005).

SREBPs are key transcription factors that play a critical role in fatty acid and cholesterol synthesis, particularly in the liver and adipose tissue. Among these, SREBP‐1c regulates the expression of enzymes required for the synthesis of cholesterol, fatty acids, triacylglycerols, and phospholipids in adipose tissue (Bertolio et al. 2019; Thering et al. 2009). In a study conducted by Mirzaei‐Alamouti et al. (2021) on sheep, the inclusion of monensin in the diet for approximately 2 months did not significantly affect *SREBP‐1c* mRNA expression in adipose tissue. Similarly, Thering et al. (2009) reported that adding fish oil or soybean oil to the diet of cattle for 21 days did not significantly alter SREBP‐1c expression in adipose tissue. However, Gallardo et al. (2015) found that lambs grazed on high‐energy pastures for 60 days exhibited elevated SREBP‐1c protein levels in adipose tissue compared to those fed on natural pastures. In this study, although *SREBP‐1c* mRNA levels showed a downregulation trend in the PG1.5 group on day 60, no significant differences were observed in the PG3 group compared to the PG0 group. Interestingly, protein levels encoded by this gene were significantly lower in the PG1.5 group than in the PG0 group. No significant differences were observed in the PG3 group for either mRNA or protein levels. This decline in the PG1.5 group could potentially be attributed to the amount of PG included in the diet. By day 90, no significant changes were noted among the groups, but on day 120, a significant reduction in SREBP‐1c mRNA levels, alongside a similar trend in protein levels, was observed in the PG3 group. These findings suggest that the decrease in SREBP‐1c activity in the PG3 group is linked to the slower adipose tissue development during this growth phase and to the high‐energy content of the diet. In agreement with these observations, de Paz et al. (2022) reported that calves fed high‐energy diets exhibited a tendency for reduced SREBP‐1c mRNA expression compared to those on normal diets, with the differences becoming non‐significant as growth progressed. Similarly, Li et al. (2019) demonstrated in bovine mammary epithelial cell cultures that lysine supplementation influenced SREBP‐1c in a dose‐dependent manner, with high‐dose lysine supplementation reducing SREBP‐1c protein levels. Consistent with these findings, the reductions observed in SREBP‐1c gene and protein levels in this study may be attributed to the dose‐dependent effects of PG supplementation. Across all experimental groups, the temporal analysis revealed similar patterns of SREBP‐1c mRNA expression, with significant downregulation on day 120 in both the PG0 and PG3 groups compared to day 60. This reduction is likely associated with the growth phase‐dependent changes in adipose tissue development.

C/EBPα plays a vital role in modulating adipokine levels and initiating adipogenesis, where it is highly expressed in adipocytes (Fonseca‐Alaniz et al. 2007). In ruminants, C/EBPα has been associated with marbling, fatty acid composition, and water‐holding capacity, serving as a candidate gene for assessing meat quality parameters (Maciel et al. 2022; Mazzucco et al. 2016; Urrutia et al. 2016; Zhang et al. 2015). For instance, Soret et al. (2016) reported that C/EBPα expression did not significantly change in subcutaneous adipose tissue during glucose‐to‐fat biosynthesis but was significantly upregulated in intramuscular fat cells in beef cattle. Similarly, Yamada and Nakanishi (2012) observed that high‐concentrate diets significantly increased *C/EBPα* mRNA levels in subcutaneous adipose tissue of cattle.

The proportion of concentrate feed and the crude protein content of the diet are among the primary factors directly influencing metabolic rate in lambs (Yang, Ahmad, et al. 2020; Yang, Wang, et al. 2020). Zhao et al. (2010) reported that a high‐protein diet alters the expression of genes involved in energy metabolism, such as SREBP‐1c and PPARγ, in adipose tissue. Similarly, Urrutia et al. (2016), in their study conducted on lambs, demonstrated that dietary composition plays a critical role in regulating lipid metabolism, thereby influencing fatty acid synthesis and deposition. These findings suggest that the variations observed in FABP4, PPARγ, SREBP‐1c, and C/EBPα gene expression and protein levels in the present study may not be solely attributed to propylene glycol supplementation but could also be affected by the energy and protein density of the diet. Although all treatment groups were fed the same ration, the relatively high metabolizable energy (2600 kcal/kg ME) and crude protein (16% CP) levels might have modulated the biological activity of propylene glycol according to differences in metabolic rate. Therefore, to better elucidate the specific effects of propylene glycol on lipid metabolism, further studies involving rations with varying energy and protein levels are warranted.

In this study, no significant differences in C/EBPα mRNA and protein levels were observed among the PG0, PG1.5, and PG3 groups on days 60 and 90. However, by day 120, both mRNA and protein levels of C/EBPα were significantly higher in the PG1.5 and PG3 groups compared to the PG0 group. This suggests that prolonged PG supplementation (120 days) influenced adipose tissue growth through C/EBPα activity. Temporal analysis indicated a significant reduction in C/EBPα expression across all groups on day 90 compared to day 60, followed by a more than 5‐fold upregulation on day 120. These findings imply that C/EBPα activity is modulated by the growth phase, influencing adipose tissue activity accordingly. Previous studies have shown significant associations between C/EBPα and lipogenesis‐related genes in lambs, further highlighting its close relationship with energy metabolism (Lôbo et al. 2012; Urrutia et al. 2016; Quiñones et al. 2017).

MCODE analysis demonstrated that SREBF1 and FABP4 exhibit strong interaction scores with key lipid‐metabolism genes, including LPL, LEP, and ADIPOQ. Moreover, these genes engage with the lipid‐homeostasis regulator NR1H3 transcription factor and its heterodimeric partner, retinoid X receptor (RXR). Network analysis further revealed close associations with additional members of the nuclear‐receptor superfamily. In bovine studies, Keogh et al. (2024) identified a significant relationship between NR1H3 expression and residual feed intake, while Basse et al. (2015) reported upregulated NR1H3 during the transition from brown to white adipose depots in sheep. Likewise, Deng et al. (2019), Xue et al. (2020) have shown that RXRA critically regulates fatty‐acid metabolism, adipocyte differentiation, and proliferation.

Our interaction data suggest that propylene glycol supplementation may modulate SREBP‐1c and FABP4 activity via RXR and NR1H3 pathways. Pathway enrichment further implicates these genes in adipogenesis, white‐adipocyte differentiation, and the white–brown adipocyte conversion process. Additionally, PPAR signaling emerged as a central axis within this interaction network. Collectively, these findings indicate that propylene glycol not only influences fatty‐acid metabolism but may also play a pivotal role in maintaining cellular homeostasis.

## 5 Conclusion

In conclusion, propylene glycol supplementation significantly regulates adipose tissue metabolism in lambs by modulating the activity of key lipogenic genes and altering the profile of fatty acids in a dose‐ and time‐ dependent manner. The results of this study suggest that propylene glycol might be an alternative effective dietary complement for fattening lambs by improving adipose tissue quality and fat composition.

## Funding

This work was supported by Türkiye Bilimsel ve Teknolojik Araştırma Kurumu.

## Conflicts of Interest

The authors declare no conflicts of interest.

## Data Availability

The data that support the findings of this study are available on request from the corresponding author.
